# Validity and reliability of a digital solution for cognitive assessment: The Brain on Track®

**DOI:** 10.1177/20552076241287371

**Published:** 2024-10-18

**Authors:** Rosa Andias, Ana Isabel Martins, Joana Pais, Vítor Tedim Cruz, Anabela G Silva, Nelson Pacheco Rocha

**Affiliations:** 1 CINTESIS.UA@RISE, School of Health Sciences, 56062 University of Aveiro, Aveiro, Portugal; 2 IEETA, Department of Medical Sciences, 56062 University of Aveiro, Aveiro, Portugal; 3 EPIUnit - Institute of Public Health, Laboratory for Integrative and Translational Research in Population Health (ITR), 26706 University of Porto, Porto, Portugal; 4 Neuroinova, Vila Nova de Gaia, Portugal; 5 Hospital Pedro Hispano (Unidade Local de Saúde de Matosinhos, EPE), Matosinhos, Portugal

**Keywords:** Cognitive assessment, Brain on Track®, community adults, validity, reliability

## Abstract

**Background:**

Cognitive assessment and the early detection of cognitive impairments have been enhanced by the use of remote digital solutions. The Brain on Track® is one of these digital solutions used in clinical practice for online screening and monitoring of cognitive functioning.

**Objectives:**

This study aimed to explore the validity and reliability of the Brain on Track® computerized test on a tablet device in adults.

**Methods:**

A community sample of 54 young adults, 51 middle-aged adults, and 50 older adults were invited to attend in two assessment sessions. The first session included data collection on sociodemographic data, user digital literacy, Brain on Track® on the computer and on the tablet device, and usability from the user and moderator perspective. The second session included the Montreal Cognitive Assessment Questionnaire (MoCA) and a second completion of the Brain on Track® on tablet to assess the criterion validity and test–retest reliability. Hypothesis testing was used to assess construct validity.

**Results:**

A weak to moderate correlation was found between the Brain on Track® tablet score and the MoCA. The ICC was 0.72, 0.84, and 0.79, and Cronbach's alpha was 0.84, 0.83, and 0.89 in young adults, middle-aged adults, and older adults, respectively.

**Conclusions:**

This study suggested that the Brain on Track® administered using a tablet device has criterion validity, particularly in middle-aged and older adults, and internal consistency and test–retest reliability in adults of any age group.

## Introduction

The integrity of cognitive performance, which includes different cognitive domains such as attention, visuospatial ability, memory, language, or executive function, is key for an individual's functional independence in daily living, and their capacity to communicate and interact with others.^1,2^ Changes in normal cognition might range from mild cognitive impairment to dementia. Mild cognitive impairment is characterized by an intermediate state of memory loss or other cognitive declines greater than expected for age and education level.^3,4^ Dementia involves severe cognitive deficits progressively impeding an individual's performance and participation.^3–5^ Age is currently identified as a major unmodifiable risk factor for cognitive impairment.^5,6^ However, other factors such as low levels of education, social isolation, smoking, physical inactivity, and comorbidities such as hypertension, obesity, and depression have been considered as relevant risk factors.^5,6^ Early detection of decline in cognitive performance, namely at an early stage of mild cognitive impairment, will enable timely therapeutic interventions, better control of potential risk factors, and potentially will delay the progression of cognitive decline, improving the quality of life of individuals and their families.^7–10^ It is also important to highlight that the detection of decline in cognitive capacity should not be restricted to older ages, as they can be observed throughout adult life and early adulthood.^11,12^ Thus, computerized cognitive assessments can offer novel benefits over traditional tests, such as allowing greater monitoring and follow-up over time.^13^

In recent years, the development and use of digital solutions have enhanced cognitive assessment and the early identification of cognitive deterioration as well as its continuous follow-up over time.^14–17^ These digital solutions provide the flexibility to assess cognitive performance at hospitals, clinics, or research laboratories, but also at home, without the need for traveling and in-person appointments.^7,8^ Some studies have demonstrated the feasibility and validity of several digital solutions in different age groups and conditions, highlighting that the interactive and user-friendly interfaces of these solutions often lead to higher individual engagement and accurate results.^16,18,19^ The correlation between digital testing and traditional in-person and paper-based assessments also underlines their clinical utility and relevance in the early detection of cognitive impairment.^9,19,20^

The Brain on Track® is an example of a digital solution used in clinical practice for online screening and monitoring of cognitive functioning.^21,22^ Brain on Track® should be completed every three months to identify longitudinal trends in cognitive performance and identify individuals with cognitive decline based on these trajectories.^23^ Its current version comprises 11 subtests that assess several domains of cognitive function (attention, memory, calculus, executive functions, language, and constructive capacity).^23^ Each cognitive domain is assessed through a set of tasks randomly generated to minimize the learning effects. The mode of interaction for each task also varies. For example, some tasks require selecting interaction (using touch or mouse click in desktop computers), while others require dragging of words or objects across the screen.^21^

Currently, the Brain on Track® is only used on the computer, the user might interact with it using the mouse, touchpad, or keyboard, and has shown good internal consistency, discriminative ability for mild cognitive impairment and early dementia, and excellent test–retest reliability.^21,23^ Additionally, a recent study explored the usability of the Brain on Track® using the computer and found good usability reported by the usability assessment moderator in a sample of community adults.^24^ However, the final score for the cognitive function was partially explained by the usability reported by the assessment moderator, suggesting that usability and patient's digital literacy skills need to be considered when using the system.^24^

In recent years, the use of touch devices, such as the tablet, has increased due to their portability and ease of interaction when compared to other solutions, such as computers.^25,26^ Furthermore, tablet devices might be easier to use by offering adult persons with limited digital literacy who may not be accustomed to using computer devices but could benefit from cognitive monitoring. However, the different mode of interaction, compared to the computer, might affect the ability and time to respond to the Brain on Track® tasks, potentially affecting its validity and reliability. Thus, the main aim of this study was to explore the validity and reliability of the Brain on Track® computerized test on a tablet device in young adults (18 to 35 years), middle-aged adults (36 to 64 years), and older adults (over 65 years).

## Methods

This validity and test–retest reliability study was approved by the Ethics and Deontology Council of the University of Aveiro (process number 39-CED/2022). Participants gave their written informed consent before entering the study.

### Sample size

The sample size calculation was based on the calculations of Walter et al.^27^ for two measurements and considering (i) an α = 0.05, β = 0.20, (ii) an acceptable Intraclass Correlation Coefficient (ICC) of 0.8 and (iii) an estimated ICC of 0.9. At least 46 participants were required for each age group.

### Participants

Through convenience sampling, young adults were recruited from universities, middle-aged adults were recruited through online community groups, and older adults were recruited from senior universities and local associations. To enter the study, participants had to meet the following inclusion criteria: (i) be 18 years old or older and (ii) be able to use a mouse/computer interface and a tablet interface autonomously. Participants with neurological, psychiatric, or systemic pathology, and/or illiteracy were excluded. The inclusion and exclusion criteria were defined by self-report.

### Procedures

Participants were required to attend two study individual sessions, within an interval of two weeks, a time interval recommended by Consensus-based Standards for the Selection of Health Measurement Instruments guidelines for studies exploring reliability.^28^ The first session included (i) data collection on socio-demographic and user digital literacy characteristics, (ii) a cognitive assessment using the Brain on Track® on the computer and on the tablet device, with a 10-min rest between the two assessments, and (iii) the completion of the Portuguese version of the System Usability Scale (SUS)^29^ to assess usability of the Brain on Track® when using the tablet device (usability from the user perspective). A trained researcher on usability assessment also completed the Portuguese version of the ICF-based Usability Scale (ICF-US; usability from the usability assessment moderator perspective).^30^ The computers and tablet devices used for data collection had similar characteristics and allowed an image flow of 20 frames per second (FPS). In this first session, half of the participants started by completing the Brain on Track® test on the tablet device, while the remaining half completed it on the computer. Subsequently, the participants switched devices.

In the second session, participants repeated the Brain on Track® on the tablet device and filled in the paper-based Montreal Cognitive Assessment (MoCA) questionnaire.^31^ Both sessions lasted approximately 60 min. Both sessions were performed in the research laboratory.

#### Socio-demographic data

Sex, age, level of formal education, current profession, and digital literacy were assessed using a purposefully developed questionnaire. Digital literacy was assessed using the following questions: (i) “Do you use the computer independently?,” (ii) “How often do you use the computer (on average)?,” (iii) “What type of interface do you regularly use to interact with the computer?,” (iv) Do you use a tablet/smartphone independently?,” (v) “How often do you use the tablet/smartphone (on average)?”.

#### Montreal cognitive assessment (MoCA)

MoCA includes the assessment of seven domains of cognitive function: (i) visuospatial capacity; (ii) executive function, (iii) attention, concentration, and working memory, (iv) language, (v) short-term memory, (vi) temporal orientation and (vii) spatial orientation.^31,32^ The different MoCA tasks can be scored from 0 to 5 points and the total scale score ranges from 0 to 30 points, with higher scores indicating better cognitive performance.^31,32^ The normative values of the scale and the cutoff points for determining cognitive decline for the Portuguese adult population vary according to age and level of education.^33^ The European Portuguese version of the MoCA was validated for three age groups (25 to 49 years, 50 to 64 years and more than 64 years) and showed good psychometric properties.^31,33,34^

#### System usability scale (SUS)

The SUS is a self-administered usability rating scale that assesses satisfaction with the use of an application or system and its ease of use from the perspective of the user.^29^ This scale consists of two subscales (i) usability (eight items) and (ii) learning (items 4 and 10). The score of each odd item is determined by subtracting “1” from the user's answer. For even items, “5” points must be added, and the value attributed to the answer given by the user must be subtracted. At the end, all items must be added, and the final value multiplied by 2.5. Thus, the total score of the scale ranges from 0 to 100 points, and higher scores indicate better levels of usability. Scores above 68 have been defined as above-average usability scores.^29^ The European Portuguese version of the SUS has good psychometric properties.^29^

#### *ICF-based usability scale* I *(ICF-Us I)*

The ICF-US I is a usability rating scale based on the evaluator's perspective, which allows a global usability assessment.^30,35^ The ICF-US I includes 10 items that assess different aspects of usability, including ease of use, level of satisfaction, task comprehension, and application responsiveness to user interaction. Each item can be scored from −3 (complete barrier) to 3 (complete facilitator). When an item is not applicable, it should be categorized as “Not applicable” (NA) and assigned the average value of the remaining items, rounded to the nearest integer. The final score of the ICF-US I is calculated by adding the scores of the scale items and ranges from −30 to 30. A value above 10 is considered good usability and less than 10 means that the technology has the possibility of usability improvement.^30,35^ The European Portuguese version of the ICF-US I has good psychometric properties.^35^

#### Brain on Track®

The Brain on Track® is a self-administered screening test that assesses cognitive performance over time.^21^ The most recent version of the Brain on Track® consists of 11 subtests, which include: (i) task 1-attention, (ii) task 2—memory 1, (iii) task 3—memory 2, (iv) task 4—calculus, (v) task 5—executive function 1, (vi) task 6—memory 3, (vii) task 7—executive function 2, (viii) task 8—language 1, (ix) task 9—language 2, (x) task 10—executive function 3, and (xi) task 11—constructive capacity.^23^ A brief description of these tasks can be found in the Supplemental Table 1.^21^ Brain on Track® tests are prescribed based on education levels, allowing the test to be adjusted to individual expected performance. The score of each subtest is based on the number of tasks performed correctly and ranges from 0 to the maximum number of tasks that the participant can perform successfully within the time limit set for the task and, therefore, has no pre-defined maximum score.^21^ Brain on Track® proved to be a valid and reliable cognitive test in the Portuguese population when applied on a computer and has been on the market since 2013.^21,23^

### Data analysis

Descriptive statistics (means, standard deviation [SD], counts (n), and percentages (%)) were used to characterize the sample. Differences between groups were explored using One-Way ANOVA for continuous data and chi-squared tests for categorical data. The first session’s differences between computer and tablet Brain on Track® scores were explored using Paired-Samples T-tests. Cronbach's Alpha (α) was used to assess the internal consistency of the Brain on Track® subtests for the tablet device. Values between 0.60 and 0.70 were interpreted as acceptable internal consistency and between 0.80 and 0.95 as very good internal consistency.^36,37^ An ICC two-way random-effects model (type 2.1) was used for test–retest reliability and interpreted as indicating: i) excellent reliability (ICC > 0.90), ii) good reliability (0.75 < ICC ≤ 0.90), (iii) moderate reliability (0.50 < ICC ≤ 0.75), or (iv) poor reliability (ICC < 0.50).^38^ Measurement error was characterized by calculating the standard error of measurement (SEM) and the smallest detectable change (SDC) using the formulae SEM = baseline SD × √(1- ICC) and SDC95% = SEM × 1.96 √2.^37^ The percentages of the SEM (SEM% = 100*SEM/mean score of the Brain on Track*®)* and SDC (SDC% = 100*SDC/mean score of the Brain on Track®) were also calculated.^39^ Criterion validity was initially assessed by correlating the Brain on Track® tablet score with the MoCA, in the second session, using Pearson's correlation coefficient (r) or Spearman's correlation (*r*_s_) depending on whether normality of data was verified. The interpretation of these correlations was (i) little or no correlation (<0.25), (ii) weak correlation (values between 0.25 and 0.50), (iii) moderate to good correlation (values between 0.51 and 0.75), and (iv) good to excellent correlation (values >0.75).^40^ Acceptable criterion validity was considered when the correlation between the Brain on Track® scores and the MoCA was ≥0.70.^40^ Additionally, considering that Brain on Track® was previously validated for the computer,^21,23^ the scores obtained on the computer and the tablet in the first session were correlated. A hypothesis for construct validity was defined based on the previous association found between usability and the Brain on Track® scores using the computer.^24^ It was hypothesized that the ICF-I and SUS would show a positive and moderate correlation with the Brain on Track® score using the tablet.

All statistical analyses were performed using the Statistical Package for the Social Sciences (SPSS) version 25 (IBM Corp., Armonk, NY, USA), and the level of significance was set at *p* < 0.05.

## Results

### Sample sociodemographic characterization characteristics

**Figure 1.**
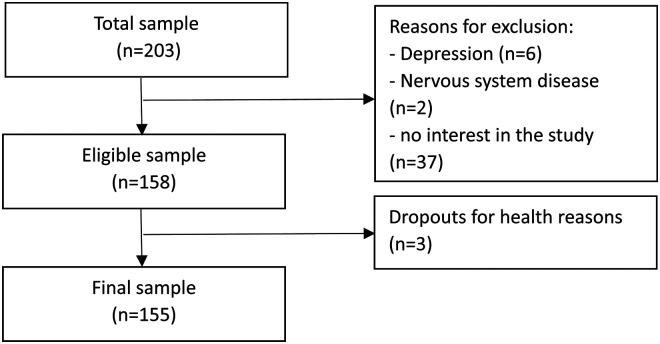
Flowchart of participants.

A total of 203 individuals were invited to enter the study. Of these 155 participants were included (Figure 1), of which 54 were young adults (mean age = 21.7 ± 4.2), 51 were middle-aged adults (mean age = 50.5 ± 9.0) and 50 were older adults (mean age = 71.7 ± 5.7). The frequency of weekly use of computer and tablet devices and other sociodemographic information can be found in Table 1. Comparisons between groups showed that (i) middle-aged and older adults groups had lower levels of education (up to 9^th^ grade) than the young adults’ group; (ii) young and middle-aged groups had a greater frequency of computer use (everyday) than the older adults group (*p* < 0.001), (iii) middle-aged and older adults groups used the mouse more frequently as an interface with the computer than the young adults’ group (*p* < 0.001) and (iv) young adults used the touchpad more frequently as an interface with the computer than the other two groups (*p* < 0.001).

**Table 1.** Sample characterization.

Variables		18–35 years old (*n* = 54)	36–64 years old (*n* = 51)	≥ 65 years old (*n* = 50)	*p*
Sex (*n*, %)	Female	44 (81.5)	34 (66.7)	32 (64.0)	0.109
	Male	10 (18.5)	17 (33.3)	18 (36.0)	
Age (years)	Mean ± SD	21.7 ± 4.2	50.5 ± 9.0	71.7 ± 5.7	<0.001
Education (*n*, %)	4^th^ grade	(0.0)	0 (0.0)	10 (20.0)	<0.001
	6^th^ grade	(0.0)	0 (0.0)	1 (2.0)	
	9^th^ grade	(0.0)	4 (7.8)	7 (14.0)	
	12^th^ grade	47 (87.0)	8 (15.7)	11 (22.0)	
	Bachelor or higher	5 (9.3)	25 (49.0)	18 (36.0)	
	Other	2 (3.7)	14 (27.5)	3 (6.0)	
Profession (*n*, %)	Salaried employee	0 (0.0)	34 (66.7)	0 (0.0)	-
	Self-employed	0 (0.0)	5 (9.8)	0 (0.0)	
	Student	54 (100.0)	1 (2.0)	0 (0.0)	
	Housewife	0 (0.0)	0 (0.0)	3 (6.0)	
	Retiree	0 (0.0)	6 (11.8)	47 (94.0)	
	Unemployed	0 (0.0)	2 (3.9)	0 (0.0)	
	Other	0 (0.0)	2 (3.9)	0 (0.0)	
Frequency of computer use^a^ (*n*, %)	Once a week	0 (0.0)	5 (9.8)	18 (36.0)	<0.001
	2 to 3 times a week	2 (3.7)	3 (5.9)	5 (10.0)	
	3 to 5 times a week	12 (22.2)	4 (7.8)	6 (12.0)	
	Everyday	40 (74.1)	39 (76.5)	21 (42.0)	
Interface^a^ (*n*, %)	Mouse	30 (55.6)	46 (90.2)	42 (84.0)	<0.001
	Touchpad	22 (40.7)	4 (7.8)	5 (10.0)	
	Touchscreen	2 (3.7)	1 (2.0)	3 (6.0)	
Frequency of tablet/smartphone use^a^ (*n*, %)	Once a week	0 (0.0)	1 (2.0)	1 (2.0)	0.072
	2 to 3 times a week	1 (1.9)	0 (0.0)	0 (0.0)	
	3 to 5 times a week	0 (0.0)	3 (5.9)	0 (0.0)	
	Everyday	53 (98.1)	47 (92.2)	49 (98.0)	

SD: Standard Deviation.

^a^ Questions used to assess digital literacy.

### Cognitive function and usability

The mean scores (±SD) on the Brain on Track®, SUS, ICF-I, and MoCA scale for the age groups can be found in Table 2. Statistically significant differences were found between the Brain on Track® scores on the computer and the tablet for all age groups in session 1, with higher scores on the tablet device (*p* < 0.05). In the three age groups, the SUS and ICF-I scores suggested good usability from the perspective of the user and of the usability assessment moderator, respectively for the Brain on Track® and for the tablet.

**Table 2.** Scores of the Brain on Track®, System Usability Scale, ICF based Usability Scale I, and Montreal Cognitive Assessment.

Variables		18–35 years old (*n* = 54)	36–64 years old (*n* = 51)	≥ 65 years old (*n* = 50)
BoT (computer)	Mean ± SD	263.4 ± 41.5	212.8 ± 42.5	140.3 ± 43.7
BoT (tablet)—session 1	Mean ± SD	276.1 ± 37.9	223.7 ± 44.6	158.9 ± 41.8
BoT (tablet)—session 2	Mean ± SD	296.5 ± 36.2	239.2 ± 40.3	167.3 ± 38.1
SUS (0–100)	Mean ± SD	89.8 ± 8.9	88.8 ± 11.4	78.2 ± 14.4
ICF-I [(−30)−30]	Mean ± SD	28.7 ± 2.3	26.6 ± 6.5	16.8 ± 9.0
MoCA (0–30)	Mean ± SD	27.7 ± 2.1	27.6 ± 2.2	25.5 ± 2.5

BoT: Brain on Track®; ICF: ICF based Usability Scale; MoCA: Montreal Cognitive Assessment; SD: standard deviation; SUS: System Usability Scale.

### Validity

#### Criterion validity

A weak correlation was found between the Brain on Track® tablet score and the MoCA score for young adults (*r *= 0.45, *p* = 0.001), as well as for middle-aged adults (*r *= 0.47, *p* = 0.001). A moderate correlation was found for older adults (*r_s _*= 0.54, *p* < 0.001).

The agreement between the Brain on Track® scores when using the tablet and when using the computer, at session 1, indicated low agreement for young adults (ICC = 0.44, [95% Confidence interval [CI]: 0.20–0.63]) and moderate agreement in middle-aged adults (ICC = 0.67, [95% CI:0.48–0.79]) and older adults (ICC = 0.55, [95% CI:0.32–0.72]). A sub-analysis for each Brain on Track® subtests can be found in Table 3. In young adults, the agreement ranged from poor to moderate (ICC = −0.01, [95% CI: −0.27–0.26] to ICC = 0.62, [95% CI:0.42–0.76]). In middle-aged, the agreement ranged from poor to good (ICC = 0.17, [95% CI: −0.11–0.42] to ICC = 0.82, [95% CI: 0.70–0.89]). In both age groups, the subtests 2 and 11 were the subtests with the lowest and highest ICC respectively. In older adults, the agreement ranged from poor to moderate (ICC = 0.13, [95% CI: −0.15–0.39] to ICC = 0.73, [95% CI: 0.56–0.84]). The subtests 4 and 8 were the subtests with the lowest and highest ICC, respectively.

**Table 3.** Correlation between Brain on Track® administered using the computer and the tablet in session 1 by subtests.

	18–35 years old (*n* = 54) ICC (95%CI)	36–64 years old (*n* = 51) ICC (95%CI)	≥ 65 years old (*n* = 50) ICC (95%CI)
Subtest 1	0.17 (−0.10 to 0.42)	0.41 (0.15–0.61)	0.54 (0.31–0.71)
Subtest 2	−0.01 (−0.27 to 0.26)	0.17 (−0.11 to 0.42)	0.38 (0.11–0.59)
Subtest 3	0.37 (0.11–0.58)	0.64 (0.44–0.77)	0.75 (0.60–0.85)
Subtest 4	0.58 (0.37–0.73)	0.54 (0.31–0.71)	0.13 (−0.15–0.39)
Subtest 5	0.19 (−0.08 to 0.43)	0.44 (0.19–0.64)	0.40 (0.14–0.61)
Subtest 6	0.49 (0.26–0.67)	0.66 (0.47–0.79)	0.52 (0.28–0.70)
Subtest 7	0.58 (0.38–0.74)	0.59 (0.38–0.74)	0.64 (0.44–0.78)
Subtest 8	0.59 (0.39–0.74)	0.77 (0.64–0.87)	0.73 (0.56–0.84)
Subtest 9	0.19 (−0.08 to 0.44)	0.39 (0.13–0.60)	0.63 (0.43–0.77)
Subtest 10	0.36 (0.11–0.57)	0.57 (0.35–0.73)	0.50 (0.26–0.68)
Subtest 11	0.62 (0.42–0.76)	0.82 (0.70–0.89)	0.68 (0.49–0.81)

ICC: Intraclass Correlation Coefficient; CI: Confidence Interval.

#### Hypotheses testing

No correlation was found between the first Brain on Track® tablet score and the ICF-I (*r *= −0.08, *p* = 0.58) and SUS (*r *= 0.06, *p* = 0.65) scores for young adults. A positive and moderate correlation was found between the Brain on Track® tablet score and the ICF-I (*r *= 0.53, *p* < 0.001), with no correlation with the SUS score (*r *= 0.05, *p* = 0.75), for middle-aged adults. A positive and moderate correlation was found between the Brain on Track® tablet score and the ICF-I (*r *= 0.42, *p* < 0.05) and SUS (*r *= 0.55, *p* < 0.001) score for older adults.

### Reliability

#### Internal consistency of the Brain on Track® for the tablet device

Cronbach's α was 0.84, 0.83, and 0.79 for young adults, middle-aged adults, and older adults, respectively, at session 1. Similar values were found at session 2 (α = 0.77, α = 0.87, and α = 0.76, respectively) (Table 4). These results suggested that internal consistency was good to very good for young adults, very good for middle-aged adults, and good for older adults, in both assessments.

**Table 4.** Internal consistency of Brain on Track® subtests in the tablet device.

Brain on Track® subtest	18–35 years old (*n* = 54)*	36–64 years old (*n* = 51)*	≥65 years old (*n* = 50)*
Subtest 1—session 1	0.83	0.82	0.83
Subtest 1—session 2	0.76	0.87	0.81
Subtest 2—session 1	0.83	0.82	0.77
Subtest 2—session 2	0.76	0.86	0.73
Subtest 3—session 1	0.84	0.82	0.79
Subtest 3—session 2	0.78	0.86	0.75
Subtest 4—session 1	0.82	0.81	0.77
Subtest 4—session 2	0.75	0.86	0.73
Subtest 5—session 1	0.81	0.80	0.78
Subtest 5—session 2	0.71	0.84	0.74
Subtest 6- session 1	0.83	0.82	0.78
Subtest 6—session 2	0.75	0.86	0.74
Subtest 7—session 1	0.83	0.87	0.80
Subtest 7—session 2	0.74	0.88	0.77
Subtest 8—session 1	0.81	0.81	0.77
Subtest 8—session 2	0.74	0.85	0.72
Subtest 9—session 1	0.83	0.80	0.74
Subtest 9—session 2	0.81	0.84	0.72
Subtest 10—session 1	0.80	0.79	0.75
Subtest 10—session 2	0.72	0.84	0.72
Subtest 11—session 1	0.83	0.81	0.78
Subtest 11—session 2	0.75	0.85	0.75
Cronbach's α total—session 1	0.84	0.83	0.79
Cronbach's α total—session 2	0.77	0.87	0.76

*Cronbach's α if the item is excluded.

#### Test–retest reliability, standard error of measurement, and minimal detectable difference

The ICC for test–retest reliability was (i) 0.72 (95% CI:0.56–0.83) in young adults indicating moderate reliability, (ii) 0.84 (95% CI: 0.74–0.91) in middle-aged adults indicating good reliability, and (iii) 0.79 (95% CI: 0.66–0.88) in older adults indicating good reliability. The SEM and SDC were, respectively, (i) 20.05 (SEM% = 7.3%) and 55.58 (SDC% = 20.1%) points in young adults, (ii) 17.84 (SEM% = 8.0%) and 49.45 (SDC% = 22.1%) points in middle-aged adults, and (iii) 19.16 (SEM% = 12.1%) and 53.11 (SDC% = 33.4%) points in older adults.

A sub-analysis of the test–retest reliability by Brain on Track® subtests can be found in Table 5.

**Table 5.** Test–retest reliability by Brain on Track® subtests.

	18–35 years old (*n *= 54) ICC (95%CI)	36–64 years old (*n* = 51) ICC (95%CI)	≥65 years old (*n* = 50) ICC (95%CI)
Subtest 1	0.42 (0.17–0.62)	0.57 (0.35–0.73)	0.91 (0.84–0.95)
Subtest 2	0.31 (0.05–0.53)	0.57 (0.35–0.73)	0.58 (0.35–0.74)
Subtest 3	0.01 (-0.26–0.28)	0.77 (0.63–0.86)	0.81 (0.68–0.89)
Subtest 4	0.73 (0.57–0.83)	0.76 (0.61–0.86)	0.34 (0.07–0.57)
Subtest 5	0.41 (0.17–0.61)	0.64 (0.45–0.78)	0.59 (0.37–0.74)
Subtest 6	0.59 (0.39–0.74)	0.59 (0.38–0.74)	0.51 (0.27–0.69)
Subtest 7	0.82 (0.70–0.89)	0.70 (0.53–0.82)	0.73 (0.57–0.84)
Subtest 8	0.77 (0.64–0.86)	0.84 (0.74–0.91)	0.70 (0.52–0.82)
Subtest 9	0.13 (−0.14 to 0.38)	0.55 (0.32–0.72)	0.65 (0.46–0.79)
Subtest 10	0.68 (0.50–0.80)	0.74 (0.58–0.84)	0.67 (0.48–0.80)
Subtest 11	0.75 (0.61–0.85)	0.82 (0.70–0.89)	0.80 (0.67–0.89)

ICC: Intraclass Correlation Coefficient; CI: Confidence Interval.

## Discussion

This study aimed to explore the validity and reliability of the Brain on Track® on a tablet device in community young adults, middle-aged adults, and older adults. Overall, the main findings of this study suggested that this digital solution seems to be valid and reliable to be administered on the tablet device in middle-aged and older adults, but there are uncertainties about its application in young adults.

Regarding the criterion validity, a weak correlation was found between the Brain on Track® tablet score and the MoCA score in young adults (*r *= 0.45) and middle-aged adults (*r *= 0.47), and a moderate correlation was found in older adults (*r_s _*= 0.54). The results of the older adults age group are in line with the results by Ruano et al.^21^ in the first validation study of the Brain on Track® administered using the computer. The Brain on Track® was administered to 39 healthy older adults (mean age = 72.2 ± 7.2) and 39 older adults with mild cognitive impairment (mean age = 73.0 ± 7.5) and the correlation with MoCA was 0.62.^21^ Despite the methodological differences found between both studies and the version used by Ruano et al.^21^ and the current version of the Brain on Track® including new subtests, the results were similar for both devices. There are no studies in middle-aged and younger adults. However, the lower correlation found in this study for the younger adults might be explained by constraints in the application of the MoCA test in the young and middle-aged adults. Firstly, although the MoCA has been validated for the European Portuguese population, with reference values established for ages over 25 years,^33^ this validation was already performed more than 10 years ago, raising the question of whether the instrument is still valid given the rapid changes in the young age groups over time particularly relating to the use of technology. This limitation might be in line with some difficulties observed in young and middle-aged adults in performing some of the tasks that are still requested in the MoCA test, namely, drawing an analog clock or performing mental calculations, which might help to explain the low associations found. Previous studies have already reported these difficulties in completing the MoCA test.^41^ Even so, overall, the young and middle-aged groups had a performance in the MoCA near the maximum score that might also have influenced the correlation. The presence of ceiling effects makes it difficult to distinguish between different levels of cognitive performance in these age groups, which, in turn, might negatively influence validity and reliability parameters. Regarding the agreement between the computer and tablet Brain on Track® global scores, a low agreement was also found for young adults, as opposed to middle-aged and older adults for whom a moderate agreement was found. The fact that younger adults use computers and smartphones more frequently and find it easier to interact and learn with digital platforms, may have influenced this correlation. Although each subtest task set is randomly and automatically generated with different elements and different combinations of these elements between tests (e.g. different words to remember, different calculations to perform) to eliminate the learning effects, the subtests of each cognitive domain are presented in the same order in all tests and the interaction with the task remains similar between tests.^23^ In this sense, even if there is no memory bias in the tasks, there is always learning about the system itself, which might have contributed to differentiate Brain on Track® performance between tests. However, it is also important to highlight that in samples that tend to be homogeneous, the ICC value tends to be lower, which may also help to explain the low agreement values found in this study.^42^

Furthermore, regardless of the device, the second administration of with Brain on Track® tends to be better than the first. A potential explanation is a learning effect that enables better interaction and interpretation of the digital solution and might have potentially influenced the correlation between tests and subtests. Again, this influence might have been more pronounced among younger adults given their heightened engagement with digital tools.^43^ The learning effect on the Brain on Track® test was also observed by Ruano et al.,^44^ in which four consecutive computer trials showed an upward trend in final scores between trials for most subtest tasks. However, it is also expected that with the repeated use of Brain on Track® over time, users will become more proficient with the system, enabling a more accurate assessment of cognitive performance as the impact from learning how to use the system decreases.^23,45^ Therefore, to maximize the utility of the Brain on Track® in clinical practice, future research needs to define how many times a user needs to use the Brain on Track® still the score stabilizes. In the present study, it seems important to highlight that the different interactions with each device, that is, the use of a mouse/touchpad on the computer and the touchscreen on the tablet, might also have influenced the ability to respond to some subtest tasks. The correlations between subtests suggest some differences, especially in younger adults in some click and drag tasks, which should be better explored in future studies in comparison with other tasks that can be facilitated by using a mouse or keyboard.

In this study, we further explored the association of the Brain on Track® performance on the tablet and the self-reported usability and the usability reported by the usability assessment moderator. At the first session, no correlation was found between the Brain on Track® scores on the tablet and usability in younger adults, but a positive and moderate correlation was found between (i) the Brain on Track® score on tablet and usability reported by the usability assessment moderator in middle-aged adults and (ii) the Brain on Track® score on tablet and the self-reported usability and usability reported by the usability assessment moderator in older adults. Overall, these results might suggest that (i) the first interaction with the Brain on Track® can assess cognitive performance without the relevant impact of the usability of the system in younger adults, and (ii) usability played a relevant role in the performance of the Brain on Track® on a tablet, in middle-aged and older adults.

Some studies have reported that younger adults use digital platforms, including in the healthcare context, much more frequently than older adults and, consequently, tend to have higher digital competence.^43,46,47^ Hence, even though this study recruited individuals from all age groups who used digital technologies in their daily routines (i.e. smartphone/tablet and computer), it is possible that younger adults, due to their improved digital literacy, found it easier to perform the Brain on Track®. Conversely, in middle-aged and older adults, the results suggest that these age groups need more time to better understand how the test works, how to interact with the system, and what instructions are requested in different tasks. These learning requirements and needs might be highlighted by the low levels of education found in this study, particularly in older adults, which highlights the real differences found in the European Portuguese elderly population. In this study, both age groups also showed greater interaction with the computer mouse, which may influence their interaction with the tablet device. Previous studies using Brain on Track® included a training session with users to contextualize them with the user interface and guarantee they understood the actions and instructions of each task.^21,23^ Furthermore, Martins et al.^24^ highlighted that individuals with lower cognitive performance show greater challenges when interacting with technology, resulting in lower levels of usability. So, individuals with lower cognitive skills and/or less digital literacy might require longer training periods to use the digital solution efficiently and minimize the impact of usability on the assessment of their cognitive performance.^21,23,24^ However, regardless of age, prior training seems essential before beginning the assessment of cognitive function, and this factor might be considered in the development of digital solutions. Currently, using the Brain on Track® as a medical device requires an initial training session that is not considered for cognitive assessment but allows the user to learn about the system and clarify doubts regarding its use.^22,23,44^ As mentioned in previous studies that used this digital solution with adults and older adults, the initial training allows the user, in a subsequent phase, to use it independently at home.

In test–retest reliability, the Brain on Track® on the tablet showed moderate reliability in young adults and good test–retest reliability in middle-aged and older adults. In the previous study that explored the test–retest reliability of the Brain on Track® with the computer, this analysis was only performed by tasks.^21^ For two consecutive trials, test–retest values ranged from 0.41 to 0.85, showing overall good test–retest reliability in adults with a mean age higher than 65 years.^21^ Additionally, the authors reported that ICC values increased using three consecutive trials (0.55 ≤ ICC ≤0.88).^21^ In the present study, a test–retest sub-analysis per task was also performed, which showed similar values in middle-aged (0.55 ≤ ICC ≤ 0.84) and older adults (0.34 ≤ ICC ≤ 0.91). In young adults, the range of ICC values was broader, ranging from 0.01 to 0.82. As reported by Ruano et al.,^21^ it was also possible to verify in the present study that the Brain on Track® ICC by subtests varies between subtests, but also by target age groups.

Comparing the results obtained on tablet and computer in this study, the total mean (±SD) scores of the Brain on Track® on computer and tablet devices were similar to a previous study that applied the Brain on Track® using the computer device.^22^ In this study of Martins et al.^22^ with 669 community-based health adults, the Brain on Track® score obtained by age groups was 279 ± 41.88 (<20 years), 264.79 ± 44.05 (20–29 years), 239.45 ± 53.04 (30–39 years), 208.17 ± 35.62 (40–49 years), 172.31 ± 45.24 (50–59 years), 151.50 ± 30.78 (60–69 years), and 145.20 ± 34.72 (70–79 years). As suggested in the present study, the results by Martins et al.^22^ also suggested a decrease in the Brain on Track® scores with increasing age, which is in line with other studies that reported a tendency to a decrease in cognitive performance as age increases.^33,48^ Furthermore, Brain on Track® scores using the tablet were higher than those obtained on the computer, which might suggest greater ease of interaction with the tablet device.

### Limitations and future research

This study has some limitations that should be considered. First, it was not possible to include an additional training session in our study methodology. However, to minimize the initial learning effect in both devices, half of the participants started by completing the test on the tablet device, while the remaining half completed it on the computer. As inclusion criteria, participants should be healthy, without neurological, psychiatric, or systematic pathology, and use smartphone/tablet, and computer interfaces in their daily routines. However, these criteria were only assessed using self-reported questions. We were unable to find an instrument validated to the Portuguese European population that assessed the digital literacy of the participants and, subsequently, allowed us to explore the impact that this variable could have on the analyses performed. Some studies have reported that health literacy levels might influence the interaction with digital applications and levels of usability.^49,50^ Further studies should continue to explore the validity and reliability of this digital cognitive test using the tablet device in other samples from different contexts and conditions, namely with persons with mild cognitive impairment and dementia and assessing sensitivity and specificity. Although Brain on Track® has already been translated into several languages, future studies should explore the validity and reliability of translated versions, as already explored for the Swedish population.^51^ In the current study, the tablet version of Brain on Track® resulted in higher scores than the computer version. Future studies should explore the need for a correction or weighting factor that allows score comparison between devices. Additionally, future studies should consider at least one training session before starting to assess cognitive performance using this digital solution. Considering this aspect, it is important to highlight that future studies should explore what number of pre-test users should perform to maximize reliability. Furthermore, considering the findings of this study, it is important to reflect on whether a system developed to assess cognitive performance at a given time for a certain population with specific characteristics might still be applied in subsequent years, in populations with other characteristics and skills/abilities.

## Conclusion

The findings of this study suggest that the Brain on Track® administered using a tablet device has criterion validity, particularly in middle-aged and older adults, and internal consistency and test–retest reliability in all age groups. More studies are needed to better explore the validity of the Brain on Track® in younger adults. Further research is needed on the properties of this digital solution using the tablet in other samples, particularly clinical samples.

## Supplemental Material

sj-docx-1-dhj-10.1177_20552076241287371 - Supplemental material for Validity and reliability of a digital solution for cognitive assessment: The Brain on Track® Supplemental material, sj-docx-1-dhj-10.1177_20552076241287371 for Validity and reliability of a digital solution for cognitive assessment: The Brain on Track® by Rosa Andias, Ana Isabel Martins, Joana Pais, Vítor Tedim Cruz, Anabela G Silva and Nelson Pacheco Rocha in DIGITAL HEALTH
