# Supplementary material for: Validity and reliability of a digital solution for cognitive assessment: The Brain on Track®
Source: Digit Health. 2024 Oct 18;10:20552076241287371. doi: 10.1177/20552076241287371 (PMC11489931; doi:10.1177/20552076241287371)
Supplement: sj-docx-1-dhj-10.1177_20552076241287371 - Supplemental material for Validity and reliability of a digital solution for cognitive assessment: The Brain on Track® [file sj-docx-1-dhj-10.1177_20552076241287371.docx]

Supplementary material

Table 1. Brief description of the Brain on Track® tasks

| Subtest | | Brief tasks description |
| --- | --- | --- |
| 1 | Attention | Two frames with geometric objects are shown and the participant should identify whether the frames are equal or different. |
| 2 | Memory 1 | Three cubes of different colors light up in a random sequence and the participant should click on the cubes in the correct order. |
| 3 | Memory 2 | The participant is asked to memorize a list of words and, after a distraction task, should click on the correct words. |
| 4 | Calculus | The participant should perform the different numerical calculations shown on the screen and enter the number via keyboard or using the mouse to click on the keyboard that appears on the screen. |
| 5 | Executive function 1 | A colored frame and the name of a color are shown on screen, and two buttons: “yes/no”. In the first set, the participant should select YES when the word and the color of the frame are the same. In the second set, the participant should select YES when the color of the frame matches the color of the word font. |
| 6 | Memory 3 | The participant is asked to memorize a list of words and, after a short pause, should click on the correct words. |
| 7 | Executive function 2 | A large arrow is shown on the screen and the participant should press the opposite keyboard arrow. |
| 8 | Language 1 | On the screen, a set of frames with geometric objects of different shapes and colors is shown and the participant should select the set that corresponds to the description of the written instruction. |
| 9 | Language 2 | The participant should select the correct category for the word that is shown on the screen by dragging the word to the corresponding box. |
| 10 | Executive function 3 | A set of figures that follows a certain logic sequence appear on the screen and the participant should select the figure that completes the sequence from 4 possible figures. |
| 11 | Constructive capacity | The screen is divided into two parts: on the right the target figure is shown, on the left several of the figure composing pieces are scattered; and the participant should drag the pieces to complete the figure. |
